# Current Strategies for Inhibition of Chikungunya Infection

**DOI:** 10.3390/v10050235

**Published:** 2018-05-03

**Authors:** Bharat Bhusan Subudhi, Soma Chattopadhyay, Priyadarsee Mishra, Abhishek Kumar

**Affiliations:** 1 School of Pharmaceutical Sciences, Siksha O Anusandhan Deemed to be University, Bhubaneswar 751029, India; priyadarsee_pharma@yahoo.co.in; 2 Institute of Life Sciences, Bhubaneswar 751023, India; abhishekbt13@gmail.com

**Keywords:** alphavirus, antiviral, chikungunya, drug likeness, drug targets, pre-clinical validation

## Abstract

Increasing incidences of Chikungunya virus (CHIKV) infection and co-infections with Dengue/Zika virus have highlighted the urgency for CHIKV management. Failure in developing effective vaccines or specific antivirals has fuelled further research. This review discusses updated strategies of CHIKV inhibition and provides possible future directions. In addition, it analyzes advances in CHIKV lifecycle, drug-target development, and potential hits obtained by in silico and experimental methods. Molecules identified with anti-CHIKV properties using traditional/rational drug design and their potential to succeed in subsequent stages of drug development have also been discussed. Possibilities of repurposing existing drugs based on their in vitro findings have also been elucidated. Probable modes of interference of these compounds at various stages of infection, including entry and replication, have been highlighted. The use of host factors as targets to identify antivirals against CHIKV has been addressed. While most of the earlier antivirals were effective in the early phases of the CHIKV life cycle, this review is also focused on drug candidates that are effective at multiple stages of its life cycle. Since most of these antivirals require validation in preclinical and clinical models, the challenges regarding this have been discussed and will provide critical information for further research.

## 1. Introduction

Chikungunya virus (CHIKV), an alphavirus of the *Togaviridae* family, is transmitted by mosquito. Following its first outbreak in 1952 in the Makonde plateau (Tanzania), it has been named after the Makonde word “kungunyala”, which means “that which bends up”, referring to the posture of patients suffering from severe joint pain during CHIKV infection. Since the reporting of its first incidence [1], CHIKV outbreaks were mostly sporadic in Africa and in Southeast Asia until 2004. In a short time, CHIKV disseminated to more than 22 countries, and its presence in the western hemisphere has been confirmed by the identification of approximately one million cases of CHIKV infection in the American continent by October 2014 [2,3]. All these events have established CHIKV as a global pathogen that continues to expand with the potential for major outbreaks.

Phylogenetic analysis of CHIKV prior to 2004 led to the identification of three distinct genotypes, including West Asian, East-Central-South African (ECSA), and African, with *Aedes aegypti* as the primary vector for transmission of CHIKV [4]. After 2004, Indian Ocean outbreaks revealed the evolution of an Indian Ocean lineage of the ECSA genotype, in which alanine at position 226 in the E1 glycoprotein was changed to valine (E1-A226V) [5]. This mutation, along with specific mutations in the E2 protein, allowed the transmission of CHIKV by an additional vector, *Ae*. *Albopictus* [5,6,7,8]. These factors led to post-2004 higher re-emergence of CHIKV. For example, Malayasia, which had limited outbreaks until 2007, experienced CHIKV epidemics with more than 10,000 cases due to infection by a mutated ECSA strain between 2008 and 2010. Unlike *Ae. aegypti*, *Ae. albopictus* has a higher capability to survive in cooler regions [9]. Thus, adaptation of CHIKV to *Ae*. *albopictus* as a vector might result in further spreading of CHIKV to tropical, as well as non-tropical, regions.

The majority of CHKV infections result in prolonged arthritis [10]. Other symptoms include myalgia, nausea, rashes [10], photophobia [11], and headaches. They also cause neurological complications [12,13]. Although the usual mortality rate of CHIKV infection is 1 in 1000 cases or less, a study on 610 patients withCHIKV infection reported 10.6% mortality and 36.4% severe morbidity [14]. The age of the patients and the presence of other comorbidities might influence the mortality associated with CHIKV infection. In recent years, CHIKV coinfection with Dengue has been reported in India [15]. CHIKV coinfection has also been reported with Dengue (DENV) and Zika viruses (ZIKV) in Colombia [16]. The morbidity and mortality associated with CHKV infection are likely to increase withan increase in the number of coinfection cases [17].

The growing emergence of CHIKV infection demands a specific antiviral strategy for minimizing morbidity and mortality. Owing to the absence of suitable vaccines and the necessity for therapeutic management of CHIKV infection, extensive research efforts have been directed towards the development of suitable antivirals. Considering the recent advances made in this field, it is worthwhile presenting an update of the antiviral development strategies that are being implemented to regulate CHIKV infection.

## 2. Current Drug Targets of CHIKV

### 2.1. Structural Proteins

The CHIKV glycoproteins, E1 and E2, constitute its icosahedral shell. While E1 facilitates membrane fusion [18], E2 helps in the binding of CHIKV to the host cell [19,20]. The binding motifs in both domains A and B of E2 facilitate interaction [21,22,23]. The third structural protein, E3, protects against premature fusion of the E2–E1 heterodimer with cellular membranes [22]. Recent studies have revealed that two amino acid residues, Gly 91 and His 230, are essential for the membrane fusion capability of E1. Any substitution at Gly 91 and His 230 positions results in loss of CHIKV E1-mediated membrane fusion [24], and researchers have hypothesized the presence of druggable pockets in these structural proteins of CHIKV. These pockets are made up of both E2 and E1 residues [25]. Hence, ligands binding to these pockets are likely to modulate E1 and E2 activity and interfere in viral fusion to the host.

Considering the multifunctional role of the capsid protein (CP), it could be an important target for CHIKV inhibition. It has two domains: the RNA binding N-terminal domain and the C-terminal protease domain [26]. While the N-terminal domain is involved in binding to the genomic RNA [27], the C-terminal domain is critically involved in the processing of structural polyprotein and the viral life cycle. Its molecular interaction with the cytoplasmic domain of E2 glycoprotein is known to facilitate the budding of virions from the plasma membrane of infected host cell [28]. Among other factors, the TF protein of CHIKV can be a potential drug target due to its involvement in viral assembly/release [29].

### 2.2. Receptors for CHIKV Entry

Other than structural proteins, several other receptors, including prohibitin (PHB), phosphatidylserine (PtdSer)-mediated virus entry-enhancing receptors (PVEERs), and glycosaminoglycans (GAGs) facilitate CHIKV internalization [30,31,32]. Receptors of the integrin family, including integrin alpha V (ITGAV) and b1 integrin (ITGB1) dimmer, also mediate virus adhesion to the cell. Accordingly, the modulation of these receptors can influence CHIKV entry in host cells [33]. CHIKV entry is also facilitated by clathrin-mediated endocytosis (CME) [34], indicating that multiple factors are involved in CHIKV internalization. Hence, blocking one factor of viral entry may not be adequate for preventing CHIKV infection. Furthermore, information on the structures of these proteins is necessary for understanding their roles prior to using them to develop CHIKV antivirals.

### 2.3. Non-Structural Protein 1(nsP1)

nsP1, a palmitoylated protein with 535 amino acid residues is involved in methyltransferase and guanylyltransferase activities, which are responsible for capping and methylation of newly formed viral genomic and subgenomic RNAs [35]. Bone marrow stromal antigen 2 (BST-2 or Tetherin) is a part of host cell defense mechanism that helps to retain the viruses at the surface of the infected cells [36]. Jones et al. showed that CHIKV nsP1 down-regulated Tetherin [37]. Thus, nsP1 can be considered as a target for developing Tetherin-mediated therapeutics against CHIKV. Interestingly, Giganteet alscreened [1,2,3]triazolo[4,5-d]pyrimidin-7(6*H*)-one against CHIKV and showed that the molecule inhibited guanylylation of alphavirus nsP1 [38]. This further validates nsP1 as a druggable target of CHIKV.

### 2.4. Non-Structural Protein 2(nsP2)

**Figure 1:**
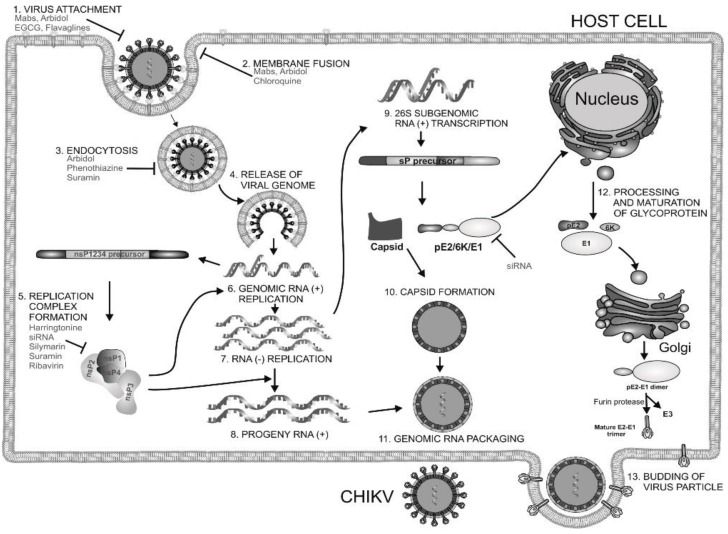
Chikungunya virus (CHIKV) replication cycle and interference by CHIKV inhibitors. CHIKV attaches (1) to the host cell, after which, it is endocytosed (3). The viral envelope and endosomal membrane fusion (2) releases the nucleocapsid (4) into the cytoplasm. The viral genome is liberated followed by translation of nonstructural proteins using the translation process of host cell, leading to the formation of viral replicase (5). This synthesizes a negative- sense RNA strand (7), which acts as a template for generation of the positive-sense RNA (8) and subgenomic (26S) RNA (9). This leads to the expression and maturation of the structural polyprotein (C-E3-E2-6K-E1) (12). The structural polyprotein is cleaved into different structural proteins and the capsid (10), which assemble with the genome to produce the nucleocapsid (11). The nucleocapsid buds out of the plasma membrane while retaining a part of the host plasma membrane with embedded glycoproteins to form the envelope of CHIKV (13). The “T-bar” represents interference in stages of CHIKV life cycle. Arbidol interferes with viral attachment, membrane fusion, and endocytosis of CHIKV. Chloroquine inhibits membrane fusion. Other agents that interfere in these early events of CHIKV cycle include EGCG, flavaglines, monoclonal antibodies, and phenothiazines. Suramin interferes both in endocytosis and CHIKV genome replication. Ribavirin, silymarin, harringtonine, and siRNA inhibit CHIKV by reducing CHIKV genome replication.

The initial viral polyproteins (Figure 1) are cleaved into individual non-structural proteins by protease activity of nsP2, which ensures productive viral replication [39]. CHIKV nsP2 is a multifunctional protein with a C-terminal proteolytic domain (cysteine protease). Its crystal structure (PDB ID: 3TRK) has been solved, and it is currently used as an important target for antiviral development. The cysteine protease is composed of 324 residues, and deprotonation of the thiol group of cysteine initiates catalysis in the active site using basic amino acids such as histidine [40]. Considering its multifunctional role, the cysteine protease is widely recognised as an important target for the development of antivirals for CHIKV. Previously, cysteine protease was reported to be papain-like, with a catalytic dyad composed of His and Cys residues [25]. However, recent observations by Saisawang et al have revealed that the cysteine protease is not papain-like, and the catalytic dyad is partially flexible [41]. Reports showed that a serine residue that is one helical turn away from the cysteine residue can be substituted while retaining catalytic activity. Catalytic activity was retained when either cysteine or serine was replaced with alanine, but substitution of both residues nullified the catalytic activity [41]. This interchangeable cysteine/serine dyad residue in CHIKV nsP2 protease currently seems to be unique, although its implications are not yet clear. Thus, further characterization of the nsP2 protease is required to successfully use it as an antiviral target. The N-terminus of nsP2 is responsible for RNA triphosphatase activity and is essential for RNA capping, assisting in the RNA helicase activity of the N-terminus [42]. Therefore, further resolution of CHIKV nsP2 crystal structure with both C-terminal and N-terminal regions might help in the development of effective antivirals for CHIKV [25].

### 2.5. Non-Structural Protein 3(nsP3)

The role of nsP3 in CHIKV replication is not clear. Nevertheless, recent reports showed that nsP3 interacts with the host stress chaperone HSP-90, which promotes CHIKV replication [43]. It has also been proposed that nsP3 blocks stress granule assembly by interacting with Ras-GAP SH3 domain-binding protein (G3BP) to promote CHIKV RNA replication [44]. Further, phosphorylation of nsP3 correlates with minus-strand RNA synthesis [45]. The N-terminal part of nsP3 contains a conserved macro domain, which may play regulatory function in CHIKV through modulation of the metabolism of adenosine diphosphate (ADP)-ribose derivatives [25]. In contrast, the C-terminal part is relatively less conserved, and hence the N-terminal domain can be considered as the active site for development of specific inhibitors of nsP3 as antivirals [46]. Since its middle region contains zinc-binding sites [47], it can also be targeted to develop potent CHIKV antiviral.

### 2.6. Non-Structural Protein 4 (nsP4)

nsP4 is RNA-dependent-RNA polymerase and a potential target for CHIKV inhibition. Accordingly, in a recent screening, a benzimidazole derivative was demonstrated to inhibit CHIKV infection by targeting nsP4 [48]. The action was demonstrated to be due to interaction with the M2295 residue in the nsP4, leading to inhibition of RNA-dependent RNA-polymerase function. It is also reported to suppress the host cell unfolded protein response (UPR) [49]. During UPR, the eukaryotic translation initiation factor 2, alpha subunit (eIF2α), is phosphorylated, leading to reduced protein synthesis and inhibition of pathogen replication. CHIKV nsP4 has been shown to inhibit this activation of eIF2α to facilitate viral translation, leading to CHIKV replication [50]. Thus, nsP4 can be targeted to develop antivirals.

Advances in basic understanding of functional roles of non-structural proteins have fuelled active research for antivirals using in silico approaches. In most of these studies, the target is limited to nsP2 protease because of unavailability of resolved structures of other non-structural proteins. Homologous modeling is an effective way of generating targeted structure, but lack of satisfactory template is a bottleneck. In this context, our group recently generated models for nsP1, nsP3, and nsP4 using the I-TASSER algorithm in order to use them as anti-CHIKV targets during antiviral development [51,52]. However, the dearth of resolved crystal structures is a major hurdle and requires further study to facilitate the development of different non-structural protein inhibitors as anti-CHIKV therapeutics. 

## 3. Hits Identified by In Silico Approaches

Hit is generally understood as a molecule with potential affinity for a specific target. Hit to lead generation occurs in the early phase of drug discovery, and involves identification of hits in high-throughput screening (HTS) in vitro, and confirmation and optimization for developing the lead molecule. The lead is expected to have good drug-likeness or pharmacological activities, which are likely to be therapeutically useful. Owing to its simplicity, Lipnski’s rule of five criteria is widely used as a guiding factor for predicting drug-likeness of compounds [53,54]. As per this rule, a molecule with not more than 5 hydrogen bond donors, not more than 10 hydrogen bond acceptors, molecular mass of less than 500daltons, and octanol-water partition coefficient not greater than 5 is expected to be orally active in humans (drug-likeness). Over the years, virtual screening has been accepted as an efficient complement to experimental HTS for identifying hits [55,56]. Elucidation of target proteins of CHIKV has encouraged this approach of identifying hits, which can be validated experimentally and optimized for finding leads and other potential antiviral candidates [57]. Amongst the proteins of CHIKV, the cysteine protease domain of nsP2 is considered as a prime target for antiviral development, mainly due to its critical role in CHIKV replication. Efforts have been made to develop antivirals targeting this molecule even before the crystal structure was available. Based on homology model of this protease, few hits have been identified that require experimental validation (Table 1, 1–3) [58,59,60]. Bassetto et al. identified some hits while working on the homology model of nsP2 protease. These compounds reduced CHIKV replication by 50% (EC_50_) at a concentration range of 3–24 μMin vitro. However, most of them also caused death of 50% viable host cells (CC_50_) at lower concentrations, leading to a narrow safety index (SI = CC_50_/EC_50_). A higher SI is always desirable for progression from in vitro to in vivo studies. Nonetheless, any SI higher than one suggests more benefits than risk [61] and can be considered for further evaluation. Accordingly, compound 3 (Table 1) with relatively better SI was considered as a hit [60]. However, subsequent studies have shown that this is an ineffective inhibitor of nsP2 protease activity [62]. This highlights the gap in extrapolation of in silico results based on homologous model. Thus, use of experimentally validated structure is more appropriate in the in silico studies for hit identification. Besides, experimental validation should be a prerequisite for progress of hit to lead. Nevertheless, structural modifications of this molecule have led to compounds that inhibit nsP2 protease activity in vitro. Amongst them, compound 53 (Table 2) exhibited good SI (>133), and this can be considered as a good hit for further optimization [62].

Availability of resolved structure of nsP2 protease (PDB ID: 3TRK) has driven further computational search to identify hits (Table 1, 4–6) [63,64,65]. Using this structure, Jadav et al. have identified a pyrazole derivative (ZINC04725220,7, Table 1)as a hit (Table 1) [100]. Recently, molecular docking studies revealed that doxycycline (8, Table 1) binds to both the catalytic domain of nsP2 and structural protein E2, which might account for its good anti-CHIKV property in vitro [66].

Unlike nsP2, other non-structural and structural proteins have received scant attention as antiviral targets in in silico studies. Earlier, Kumar et al. developed a homology model of nsP4 and identified BILN2106 and JTK 109 (9–10, Table 1) as two potential hits through molecular docking studies [67]. Recently, our group identified MBZM-N-IBT (Table 2) as a good hit, with affinity for the non-structural and structural proteins. This was supported by its potent inhibition of CHIKV nsP2 and E1 expression levels in vitro [52]. Molecular docking analysis showed affinity of baicalin, naringenin, and quercetagetin (11–13, Table 1) towards nsP3 (PDB ID: 3GPG) [101], although this is yet to be experimentally validated. Nonetheless, naringen ininhibited renilla luciferase marker gene activity in the CHIKV replicon [69] and can be hence considered as a hit against CHIKV. Lack of valid structure is also a hurdle in use of structural proteins for structure-based drug design to develop antivirals against CHIKV. Nevertheless, using a homologous model of capsid protein of CHIKV, picolinic acid (14, Table 1) was identified and validated as an antiviral hit against CHIKV [70]. These hits need to be investigated against specific targets of CHIKV. However, currently, availability of resolved target is a challenge. Hence, purification of these proteins and assaying of their activity will boost experimental confirmation of hit molecules, which can then be developed as potential leads against CHIKV.

## 4. Drugs Inhibiting CHIKV Entry

### 4.1. Chloroquine

Since 1934, chloroquine (1, Table 2) has been known as an antimalarial drug. Its repurposing against viral infections has been studied because of its ability to inhibit the pH-dependent steps of flavivirus, retrovirus, and coronaviruses replication [102]. Additionally, it has immunomodulatory effects, which alleviate the inflammatory complications of viral diseases [102]. Similar to the results of these studies, Khan et al. (2010) [71] reported inhibitory effect against CHIKV within 1–3 h post-infection (hpi) and ineffectiveness in late stages of infection (Figure 1) [71]. Inhibition at early stages of CHIKV infection is attributed to increase in the endosomal pH, which prevents E1 fusion during CHIKV internalization [103]. These reports highlight the suitability of chloroquine use for prophylaxis, albeit with limited therapeutic utility. This is evident from both in vivo experiment and clinical trials, where chloroquine was ineffective in post CHIKV-infected cases [104]. The benefits of chloroquine include its capacity to reduce symptoms of arthritis, which is a major cause of CHIKV-related morbidity [105]. However, recent studies in India showed that it does not offer better benefits than non-steroidal anti-inflammatory drugs (NSAID), including meloxicam, in arthritis following acute CHIKV infection [106].

### 4.2. Arbidol and Its Derivatives

Arbidol (2, Table 2), also known as umifenovir, was invented through collaborative research by Russian scientists from the Chemical-Pharmaceutical Scientific Research Institute of Russia, the Scientific Research Institute of Medical Radiology in Obninsk, and the Leningrad-Pasteur Scientific Research Institute for Epidemiology and Microbiology. Its clinical efficacy was first reported as a prophylactic agent against respiratory diseases caused by influenza A and B viruses [72,73]. Since 2006, it has been marketed in Russia and China for prophylaxis and treatment of viral pulmonary disorders. Further, in vitro efficacy of arbidol has been demonstrated against a wide range of viruses, including hepatitis C virus (HCV), Ebola virus (EBOV), Tacaribearena virus, and human herpes virus 8 (HHV-8) [107]. Its good in vitro inhibitory activity (EC_50_: 12 µM), along with higher SI (>15) against CHIKV (Table 2), was reported by Delogu et al. (2011) [108]. In this work, a CHIKV mutant resistant to arbidol was shown to have a single amino acid substitution (G407R) localized in the E2envelope protein. Arbidol is suggested to interfere during attachment and entry of CHIKV into host cell. CHIKV replication complexes form type 1 cytopathic vacuoles by attaching to the membrane of endosomes and lysosomes. This produces viral RNA, leading to CHIKV replication (Figure 1).Owing to its hydrophobicity, arbidol is incorporated into the membrane to alter the formation and integrity of these vacuoles, leading to inhibition of CHIKV replication [36]. In spite of their efficacy in vitro, information on their clinical efficacy against CHIKV is limited. Effectiveness only at early stages of CHIKV life cycle raises questions about its therapeutic efficacy. Accordingly, efforts have been made by Di. Mola et al. to develop more potent arbidol derivatives against CHIKV [74]. Amongst these, compounds 3 and 4 (Table 2) exhibited better SI and higher potency than arbidol. In contrast to the results of previous reports, a lower SI of 4.6 was reported for arbidol in this investigation. This variation can be due to the different cells used in the in vitro studies. Further determination of CC_50_ values in multiple relevant human cell types can be a better guiding factor. In addition, the mode of action requires detailed investigation prior to further evaluation. Nonetheless, while abiding by Lipinski’s rule of five [109], these compounds can be evaluated in pre-clinical models to determine their effectiveness.

### 4.3. Phenothiazines

CME is one of the ways by which CHIKV is internalized in the cytoplasm [34]. Chloropromazine (5, Table 2), a phenothiazine drug, is known to block the formation of clathrin-coated pits [110], and therefore it can be expected to prevent viral entry. Indeed, Pohjala et al. [69] have utilized the above potential of this class of compounds against CHIKV and Semliki forest virus (SFV). Using SFV as surrogates, they have suggested that chloropromazine, thiethylperazine, thioridazine, perphenazine, ethopropazine, and methdilazine (EC_50_: 11.3 μM to 25.1 μM) might interfere with CHIKV entry. While these compounds did not show any effect on CHIKV replicon formation, they inhibited CHIKV-Rluc infection. This was in agreement with the demonstrated capacity of these compounds ininhibiting SFV entry into BHK cells [69]. Although, the exact mechanism is yet to be elucidated, they are suggested to reduce CHIKV entry by reducing CME. However, CHIKV is also capable of entering cellsvia clathrin-independent pathways [34]. Hence, blocking of CME alone may not be adequate for reducing CHIKV infection. In addition, phenothiazines are central nervous system (CNS) drugs, and therefore any repurposing of these drugs for clinical benefits against CHIKV should also consider their CNS pharmacodynamics.

### 4.4. Epigallocatechin Gallate (EGCG)

Most of the primary attachments of human viruses are mediated through either interaction between basic binding pockets in the virion glycoproteins and negatively charged heparan sulfate moieties in cellular glycosaminoglycans or interactions with sialic acid-containing sialoglycans. Capitalizing on these mechanisms, receptor mimetics have been identified to interfere with virus entry [75,111]. While many of these either bind to heparan sulfate or sialic acid, for the first time, Colpitts et al. showed that epigallocatechingallate (EGCG) interferes with both pathways of virus entry [112]. This mode of action has been suggested against several unrelated viruses, including HSV-1, HCV, and IAV [111,112]. Prompted by this, Weber et al. screened EGCG (6, Table 2) against CHIKV and observed a 60% reduction in infection rate at a concentration of 10 μg/mL [113]. EGCG significantly inhibited transduction with CHIKV Env-pseudotyped lentiviral vectors. Furthermore, evidence of its interference in CHIKV entry was obtained by analyzing its influence on cell attachment of CHIKV. It reduced the number of infected cells at an MOI (multiplicity of infection) 1 and 10 [112]. However, it failed to completely block the entry of CHIKV; its ability to prevent CHIKV entry can be considered encouraging, and further exploration of its effect on CHIKV replication is necessary. However, clinical efficacy of EGCG is of concern because of poor oral pharmacokinetics [114]. Hence, the in vivo efficacy of EGCG against CHIKV is likely to be a major challenge for its further exploration as an antiviral.

### 4.5. Flavaglines

Flavaglines are natural products from plants of the genus *Aglaia*. Since reporting of the anticancer effect of the first member of its family (rocaglamide), about 50 other flavaglines have been identified [115]. As a group, they are endowed with several bioactivities, including anticancer and antiviral properties [53,116]. Encouraged by the identification of prohibitin-1 (PHB-1) as a receptor protein for CHIKV entry into mammalian cells [30] and reports of ligand properties of flavaglines for PHB [117], Wintachai et al. screened synthetic flavaglines, including FL3 (7, Table 2), against CHIKV [76]. FL3 was reported to be the most potent amongst test compounds for reducing CHIKV infection when pre-incubated (1 h) with HEK 293 T/17 cells. In contrast, negligible reduction in CHIKV infection was observed with the addition of these compounds post-infection. This indicated interference of FL3 in CHIKV entry, which was confirmed by significant reduction of PHB1 in the co-localization study [76]. However, this effect was observed in the entry of about 50% CHIKV, which again highlights the fact that CHIKV uses multiple ways of cell entry, which results in limited prophylactic effect of these compounds. Nevertheless, FL3 can be a good hit for further optimization considering its drug likeness and agreement with Lipinski’s rule [109]. This is also supported by the fact that it is not cytotoxic in vitro [76] and exhibits cardio protective properties in vivo [118].

### 4.6. NSAIDs

Non-steroidal, anti-inflammatory drugs (NSAIDs) are administered to manage the arthritic symptoms of CHIKV infection. Methotrexate and sulfasalazine have also been used to manage inflammatory polyarthritis following CHIKV infection [119]. Interestingly, in a recent study, the acidic class of NSAIDS consisting of anthranilic acid derivatives, including mefenamic acid, meclofenamic acid, flufenamic acid, and tolfenamic acid, showed direct evidence of antiviral action against CHIKV. Mefenamic acid and meclofenamic acid (8, 9, Table 2) exhibited an EC_50_ of 13 μM and 18 μM, respectively, with CC_50_ more than 100 μM in Vero cells [66]. While these NSAIDS inhibited CHIKV at the entry level by some undefined interaction with CHIKV envelope, there was little effect on CHIKV replication. To determine whether these NSAIDS can complement the CHIKV replication inhibitory capacity, combinations (1:1) of mefenamic acid and meclofenamic acid with ribavirin were investigated, which showed higher antiviral potency with EC_50_ of 3 μM and 5 μM, respectively. This in vitro synergistic antiviral action of ribavirin with mefenamic acid (1:1) was further validated in vivo, which showed a 6.5 fold reduction in CHIKV titer [66]. Additionally, pathological signs were significantly reduced by this combination, which was ascribed to a combination of the antiviral and anti-inflammatory effects of mefenamic acid [66]. Considering these findings, mefenamic acid can be considered as a useful drug to repurpose against CHIKV. However, when used alone, its therapeutic efficacy can be expected to be low because of its limited capacity to inhibit CHIKV replication. In this respect, its benefits can be compared to those of chloroquine, as both drugs antagonize CHIKV entry [120]. Nonetheless, it can be administered in combination with antivirals such as ribavirin, which act at post-entry levels of CHIKV. While the synergistic antiviral effects of drug combinations are desirable, other pharmacodynamic consequences must be studied in detail before further clinical trials.

### 4.7. Imipramine 

Phalenet al. have identified a central role of cholesterol in alphavirus infection [121]. Accordingly, pre-treatment with a potent cholesterol-depleting agent, methyl β-cyclodextrin (20 mm), was shown to reduce CHIKV infection by 63% [103]. Encouraged by this, recently Wichit et al. investigated cholesterol trafficking inhibitors, including U18666A (Table 2) and imipramine, against CHIKV infection in primary human epidermal fibroblasts [77]. The CHIKV entry inhibitory capacity of U18666A (10, Table 2) was similar to the effects observed earlier against DENV [71] and Ebola virus [65]. Imipramine (11, Table 2) inhibited the entry and impaired the post-fusion viral RNA replication steps, suggesting interference in distinct steps of CHIKV infection cycle with a key role of cholesterol. It was also found to be effective against different arboviruses, including DENV, West Nile virus (WNV), and ZIKV [68]. In addition, the ability to inhibit filovirus entry and infection are indicative of the broad spectrum action of imipramine [51]. Desipramine, an imipramine metabolite, is also capable of blocking cholesterol transport and may show similar potential against CHIKV [120]. Since imipramine is approved for human use, it can be prioritized for clinical validation. However, it is an anti-depressant drug capable of affecting neurotransmitter systems and requires careful consideration of benefits to risk ratio. Moreover, other cholesterol inhibitors, including dynasore [122], ezetimibe [108], and statins, have earlier shown antiviral effects, and therefore, their effects on CHIKV will be of particular interest.

### 4.8. Monoclonal Antibodies

The potential of antibodies in controlling CHIKV infection has been suggested by several groups [123,124]. Following isolation of CHIKV neutralizing antibodies (NAbs), several research groups have demonstrated their ability to protect mice and non-human primates against CHIKV infection [94,97,125,126]. In most of these studies, monoclonal antibodies (mAbs) were shown to block the virus only at the entry stages of CHIKV infection in vitro. However, in a recent study, CHIKVNAbs were shown to prevent CHIKV entry, as well as release. The C9 [125] and IM-CKV063 [94] NAbs were shown to block entry and release of CHIKV in vivo. Interaction with the domain A and the β-ribbon connector of one E2 and domain A and B of a neighboring E2 was proposed to affect the fusion process, leading to prevention of CHIKV entry [96]. IM-CKV063, which has higher potential, should be investigated as a candidate for monotherapy against CHIKV infection.

### 4.9. Curcumin

Curcumin (12, Table 2), the major active compound in the rhizome of turmeric (*Curcuma longa*), possesses antiviral properties against a range of viruses [127]. Recently, it was reported to be effective against CHIKV [78,128]. It showed an IC50 of 3.89 µm against CHIKV. However, with a CC50 of 11.6 µm, the safety index was not satisfactory. Nonetheless, its derivative (demethoxycurcumin) showed higher potency and better safety profile (13, Table 2). Owing to its lipophilic properties, demethoxycurcumin can interfere with the CHIKV membrane, which contributes to its antiviral action. Although the in vitro efficacy of curcumin has been reported against many infectious diseases, the effect was not translated in vivoin most cases owing to poor aqueous solubility and bioavailability [129]. Thus, the in vivo efficacy of curcumin derivatives requires validation prior to its further use. Nonetheless, it can be derivatized to optimize its pharmacokinetics and enhance its anti-CHIKV properties.

## 5. Inhibitors of Viral Genome Replication

### 5.1. Andrographolide

*Andrographispaniculata* has been traditionally used in southern and southeastern Asia to treat different infectious disorders. Considering the antiviral properties [130,131,132] of andrographolide (14, Table 2), Wintachai et al. screened it against CHIKV [79] and observed that it reduced CHIKV replication with an EC_50_ of 77 μM and good SI (>14) (Table 2). It was shown to interfere in the post-entry step of CHIKV replication [79]. Results of a proteomics study indicated that the anti-CHIKV property was mediated by interaction with actin [133]; however, in the experimental set up, it did not alter actin expression [79]. Nevertheless, significant reduction in RNA and protein levels implied that andrographolide directly targeted either the CHIKV genome or proteins [79]. Considering its wide range of effects, multiple modes of antiviral actions cannot be ruled out. While in vitro screening shows promise of antiviral activity against CHIKV, its in vivo efficacy needs to be established. Since, oral andrographolide suffers from limitations, including inadequate stability in gastrointestinal tract coupled with poor absorption and rapid clearance, further optimization is required to demonstrate efficacy in pre-clinical models [134].

### 5.2. Ribavirin

Ribavirin (15, Table 2) is a broad-spectrum antiviral, and its use has been approved against respiratory syncytial virus infection in infants [135] and chronic hepatitis C virus infections [136]. It also showed good antiviral activity against CHIKV (EC_50_: 15.51 ± 1.62 µM) and exhibited synergistic effect with doxycycline (EC_50_: 10.95 ± 2.12 μM) when combined using a 1:1 ratio (EC_50_: 4.52 ± 1.42 μM) in vitro [80]. Similar synergistic activity was also reported with IFN-α2b [82]. Multiple pathways have been postulated for its antiviral action. Inhibition of inosine monophosphate dehydrogenase (IMPDH), leading to depletion of GTP pools, as well as inhibition of viral RNA capping, are considered as the major routes via which ribavirin exerts antiviral action against CHIKV [136]. However, recently, our group demonstrated that ribavirin is effective only in the early stages of CHIKV life-cycle, which may be a disadvantage regarding its therapeutic efficacy against CHIKV [52].

### 5.3. Mycophenolic Acid (MPA)

MPA (16, Table 2), a synthetic antibacterial molecule, was discovered in 1893. This drug was approved in 1995 (USFDA) for preventing organ transplant rejection. MPA was earlier shown to be effective against a range of unrelated viruses, with antiviral activity being reversed by administration of guanine type of compounds [137]. This indicated their ability to interfere in viral nucleic acid synthesis, and now they are recognized as non-competitive inhibitors of inosine-5′-monophosphate dehydrogenase (IMPDH) [138]. It is also reported to act against dengue virus by interfering in viral replication via similar mode of action [139]. Considering its inhibitory action on IMPDH, Khan et al. assessed the effect of MPA on CHIKV replication (Table 2) [81]. At a non-toxic concentration (10 μM), MPA reduced more than 99.9% CHIKV titer in Vero cells. It was also observed to be more potent in depleting intracellular GTP-polls than ribavirin [81]. However, its benefits must be considered in the wake of its toxicity as an immune-suppressant drug, which can pre-dispose the users to opportunistic infections [140]. In addition, Rashad et al. failed to reproduce the CHIKV inhibitory actions of MPA or its related derivatives [141], which raises serious questions regarding further use of MPA as a lead molecule for developing anti-CHIKV therapies.

5.4. 6-Azauridine

6-Azauridine (17, Table 2) was reported as an anti-metabolite and was clinically effective against psoriasis [142,143]. It was further reported to possess broad spectrum antiviral activity against DNA and RNA viruses as its metabolite, 6-azauridine-5′-monophosphate, blocked orotidylic acid decarboxylase [144]. Later, it has also been shown to effectively reduce CHIKV-induced cytopathogenicity in vitro (SI = 204) and was reported to be more potent than ribavirin (Table 2) [82]. This antiviral action against CHIKV has also been further validated by independent studies, in which the EC_50_ value was in the range of 1–6 μM [69,145]. Being a nucleoside analogue, it was proposed to interfere with cellular UTP metabolism by incorporating into CHIKV RNA, leading to ‘error catastrophe’ [145] and cell death. Considering its good oral bioavailability and history of use without serious toxicity, 6-azauridine needs to be evaluated in animal models [142,143]. Earlier reports have shown that it causes hemorrhagic diarrhea, leading to high mortality in rats, whereas it is well tolerated by pigs [146]. Since pigs are not suitable models for CHIKV infection because of their tolerability for this virus, selection of a suitable model for its pre-clinical evaluation may be a bottleneck for further investigations.

### 5.5. Favipiravir (T-705)

T-705 (18, Table 2) is a pyrazine carboxamide derivative that is effective against a wide range of RNA viruses [147,148]. In 2014, it was approved in Japan for stockpiling against influenza pandemics. It acts as a prodrug and gets phosphoribosylated by cellular enzymes to its active form, favipiravir-ribofuranosyl-5′-triphosphate. In this form, it competitively inhibits the incorporation of ATP and GTP by the viral RNA-dependent RNA polymerase (RdRp), leading to chain termination [149]. This unique mode of action against RNA viruses has encouraged its screening against some laboratory strains and clinical isolates of CHIKV in vitro, where it has been found to effectively reduce CHIKV replication (Table 2) [83]. Delang et al. also reported the efficacy of T-1105 (19, Table 2), the defluorinated analogue of favipiravir, in inhibiting CHIKV replication (Table 2). Interestingly, a K291R (in nsP4) mutant of CHIKV developed resistance against favipiravir and its analogue, T-1105 [83]. In view of thein vitro synergistic interaction of flavipiravir with ribavirin against Lasavirus [150], it will be interesting to see if similar effects can be observed against CHIKV.

### 5.6. RNA Interference Targeting CHIKV Genes

Small interfering RNAs (siRNA) are double stranded RNA molecules that interfere with gene expression. They affect translation by degrading mRNA after transcription, which makes them potential antiviral agents against RNA viruses, including CHIKV. In agreement with this, siRNAs have been shown to reduce CHIKV titers by 99.6% by targeting CHIKV genes encoding nsP3 and E1 at 24 hpi [58]. However, at 72 hpi, it was unable to sustain the same efficacy due to both intracellular degradation and rapid viral replication. This is also evident from inhibition of CHIKV replication in CHIKV-infected Swiss albino and C57BL/6 mice, in which administration of siRNA at 72 hpi completely reduced CHIKV infection [151]. Similarly, short hairpin RNAs (shRNAs) against CHIKV E1 and nsP1 genes significantly reduced CHIKV infection compared to the moderate inhibition by shRNAs targeting the capsid [152]. E1-shRNA was effective against several CHIKV strains, but did not inhibit other RNA viruses, including dengue and Sindbis viruses, indicating their specificity for CHIKV [152]. However, intracellular degradation still remains a hurdle in its application [153]. Recently, Lam et al. (2010) showed protective effect of phosphorodiamidatemorpholino oligomer (PMO) against CHIKV [154]. Two anti-CHIKVPMOs (CPMO), CPMO1 and CPMO2, selectively interfered with the CHIKV RNA genome without inducing cytotoxicity. The PMOs reduced CHIKV titer and lowered E2 protein expression, while preventing CHIKV-induced CPE [154]. The efficacy and stability of these PMOs provide a good case for investigating them as anti-CHIKV therapeutics.

### 5.7. Silymarin

Silymarin, a complex of flavonoids, including silydianin, silychristin, and isosilybin from the plant *Silybummarianum*, is used to manage liver diseases owing to its antioxidant properties [155]. In addition, it is used as a potent therapeutic against chronic viral hepatitis because of its ability to prevent HCV entry and transmission [156]. A recent report showed that silymarin significantly inhibited CHIKV (EC_50_:16.9 μg/mL; CC_50_: 425.1 μg/mL). Unlike silymarin, other flavonoids, including quercetin and kaempferol, did not show significant CHIKV inhibition under the same experimental condition. Silymarin was most effective when administered at 2 hpi. In contrast to its antiviral action at the level of HCV entry into host cells, silymarin interfered with the post-entry stages of CHIKV replication. Significant inhibition of protein synthesis was attributed to the reduction in CHIKV RNA replication [157]. It is also possible that some of these proteins are direct targets of silymarin, a proposition that requires further investigation. Considering its safety, reasonable bioavailability, and antioxidant properties, silymarin can be a good candidate for in vivo evaluation [155]. However, the contribution of the individual constituents of silymarin to anti-CHIKV properties is not clear. Herbal drug components vary and need to be authenticated using combinatorial markers before being evaluated as “herbal drug” [158]. Thus, antiviral quality control by standardization and validation of constituents prior to further approval as herbal drug will be a major challenge. 

### 5.8. Suramin

Suramin (20, Table 2) has been approved by the FDA for the treatment of trypanosomiasis in humans. In addition, it possesses both anticancer and antiviral properties. Recently, it was shown to be effective against CHIKV in vitro (Table 2) [159]. Against different CHIKV strains, it showed EC_50_ values in the range of 8.8 μM to 28.9 μM, with a CC_50_ value of more than 700 μM. Suramin inhibited the early progression of CHIKV, suggesting interference in CHIKV entry. Studies by Albulescu et al. also supported the role of suramin at early stages of CHIKV life cycle. Additionally, it also showed strong potential to inhibit CHIKV RNA synthesis (EC_50_: 5 μM) in vitro [84]. CHIKV (nsP4) mutants did not exhibit cross-resistance to suramin. These mutants were resistant to RNA inhibitors, including favipiravir or ribavirin, but susceptible to suramin. Hence, the efficacy of suramin against CHIKV may be due to some other mode of action, which is currently not understood. Thus, suramin possibly mediates its anti-CHIKV properties by multiple pathways, including inhibition of RNA synthesis, and can be a good hit for further pre-clinical and clinical evaluation against CHIKV. Recently, Hwu et al. developed 20 structural analogues of suramin [160]. Although few compounds showed encouraging results, their benefits are not clear at present due to lack of enough evidence on their mode of interference, and further optimization is required to improve their acceptability. In addition, other attempts have not yielded more suitable derivatives of suramin [159]. Interestingly, synergistic CHIKV inhibitory activity was demonstrated between suramin and EGCG [161] in a recent drug screen. Considering their multiple modes of interference, the exact nature of this complementary action is not clear. Nevertheless, it highlights an alternative strategy of inhibiting CHIKV infection following further in vivo validation. 

## 6. Inhibitors of Viral Protein Translation

### Harringtonine and Homoharringtonine

Using an immunofluorescence-based approach, Kaur et al. screened a library of natural compounds and identified hits for anti-CHIKV molecules. Among them, harringtonine (21, Table 2), hypocrellin A, rottlerin, and daunorubicin inhibited CHIKV in a dose-dependent manner. Further studies revealed harringtonine to be a potent antiviral (EC_50_ of 0.242 µM) against CHIKVin vitro with minimum cytotoxicity (Table 2) [85]. Studies demonstrated that the early events of the CHIKV replication cycle were inhibited after viral entry into cells without affecting CHIKV binding and entry. Since harringtonine decreased CHIKV RNA production and synthesis of the nonstructural (nsP3), as well as structural (E2) proteins, it was suggested that it interfered with protein translation. Homoharringtonine, an analogue of harringtonine, also inhibited CHIKV replication. Despite its lower protein synthesis inhibitory ability than homoharringtonine, harringtonine showed stronger CHIKV inhibition [85,162]. Thus, other pathways of CHIKV inhibition cannot be ruled out in addition to inhibition of CHIKV protein translation as possible mechanisms of harringtonine and homoharringtonine action. The in vitro action of harringtonine against CHIKV infection of primary human skeletal myoblasts, an in vivo target of CHIKV, suggests its potential as an antiviral hit. However, the Globally Harmonized System (GHS) (https://www.unece.org/fileadmin/DAM/trans/danger/publi/ghs/ghs_rev04/English/ST-SG-AC10-30-Rev4e.pdf) categorizes it to be fatal when swallowed (H300). Hence, detailed optimization may be essential to further its utility against CHIKV.

## 7. Host-Targeting Antivirals

### 7.1. Furin Inhibitors

Host cell proteases, including furins, have been implicated in the cleavage of surface glycoprotein E3/E2 and production of mature virions of alphaviruses [163]. Membranous furin has been shown to process E3/E2 from African CHIKV strains by acting on HRQRR^64^↓ST motif at the E3E2 junction. On the contrary, a CHIKV strain of Asian origin is cleaved at the RRQRR^64^↓SI site by membranous and soluble furin [62]. Thus, blocking of these cellular convertases is a viable approach for preventing formation of mature CHIKV particles. Indeed, Ozden et al. demonstrated that decanoyl-RVKR-chloromethyl ketone (dec-RVKR-cmk,22, Table 2) significantly inhibited CHIKV infection in human muscle satellite cells because ofits capacity to irreversibly block furin [62]. It showed a stronger ability to reduce CHIKV infection than chloroquine when it was added immediately after infection. Interestingly, it showed additive effect with chloroquine, supporting the fact that these molecules have different modes of action. While chloroquine prevented viral entry, dec-RVKR-cmk prevented maturation, leading to near-suppression of CHIKV infection (Table 2). In addition, dec-RVKR-cmk was also suggested to alter the processing of proteins involved in CHIKV endocytosis, indicating arole in preventing CHIKV entry [62]. Notwithstanding the antiviral effects of furin inhibitors, their applicability is a major issue because of the presence of other furin substrates, including transforming growth factor beta 1 precursor, membrane type-1 matrix metalloproteinase, and the beta subunit of pro-nerve growth factor, which play important roles in normal physiological processes. Thus, selectivity and toxicity are major bottlenecks in the clinical application of furin inhibitors, while stability associated with the large size of the furin inhibitors also limits their therapeutic utility [164]. Thus, benefits and risk evaluation in pre-clinical models may reveal its utility for further application. 

### 7.2. Protein Kinase C (PKC) Modulators

Cellular activation leading to up-regulation of viral expression, followed by its immune clearance, has been used as a strategy against human immunodeficiency virus (HIV) to reduce the latent reservoir of the virus. The protein kinase C (PKC) pathway up-regulates latent HIV-1 expression, and molecules, including phorbol esters and certain lactones, have been screened against HIV as PKC modulators [165]. Accordingly, this strategy was investigated against CHIKV by many researchers. Prostratin (23, Table 2) and 12-*O*-tetradecanoylphorbol 13-acetate (TPA, 24, Table 2) are tagline diterpenoids, reported to act as PKC modulators and CHIKV replication inhibitors in Vero cells [86]. TPA possessed higher potency (EC_50_: 3 nM) with an SI close to 2000. Its efficacy was specific to CHIKV, as it did not affect other alphaviruses, including SINV and SFV [86]. In a recent report, TPA and 12-*O*-decanoyl-7-hydroperoxy-phorbol-5-ene-13-acetate (25, Table 2) were reported as the most potent components of an ethyl acetate extract of the leaves of *Croton mauritianus* with EC_50_ of 2.4 ± 0.3 and 4.0 ± 0.8 μM, respectively, against CHIKV in vitro (Table 2) [87]. Aplysiatoxin derivatives are known to activate PKC [166]. Recently, two such compounds, debromoaplysiatoxin (26, Table 2) and 3-methoxydebromoaplysiatoxin (27, Table 2), obtained from the marine cyanobacterium, *Trichodesmiumerythraeum*, were reported to inhibit CHIKV. Their debromo analogues showed significant anti-CHIVK activities (EC_50_: 1.3 and 2.7 μM) in vitro [88]. Phorbol-12, 13-didecanoate (28, Table 2), with an EC_50_ value of 6.0 ± 0.9 nM against CHIKV, was also proposed to mediate its antiviral properties through PKC [89].

Recently, bryostatin analogues were reported to exhibit high potency and low toxicity against CHIKV based on their ability to modulate PKC [167]. Interestingly, bryostatin 1, which is a potent pan-PKC modulator, was inactive against CHIKV in the same assay. This led to the suggestion that the activities of these molecules are also mediated by a PKC-independent pathway. Furthermore, evidence regarding PKC-independent anti-CHIKV activity for bryostatin analogues was provided by the same group in a study of salicylate-derived bryostatin analogues against CHIKV [90]. One of these analogues (bryostatin-21, 29, Table 2) inhibited CHIKV (Table 2) without modulating PKC with high potency (2.2 μM) and good SI (>22.72). While these findings suggest PKC-independent pathways for cell protection, they do not rule out PKC as an important target for protection against CHIKV. Although these studies suggest the prospect of PKC modulators as antivirals for CHIKV, their clinical side effects require careful consideration before developing them further as antivirals. This is because different PKC isoforms play crucial roles in normal physiology, and their non-selective inhibition may limit the benefits of using them as antivirals [168]. 

### 7.3. Kinase Inhibitors

In an attempt to identify hits against CHIKV, Cruz et al. randomly screened a library of kinase inhibitors against CHIKV infection in vitro and identified six compounds with structurally diverse features, including a benzofuran, pyrrolopyridine, and thiazol-carboxamide [91]. Amongst the hits identified, compound 30 (Table 2) was found to be most potent with high SI. These compounds were proposed to interfere with CHIKV-induced CPE formation through the inhibition of kinases involved in apoptosis. However, their specificities against other kinases require further investigation. Recently, sphingosine kinase 2 (SK2) was identified as a selective CHIKV host factor that co-localized with CHIKV replication complex during infection [169]. Thus, selective inhibition of SK2 can be an important way to determine the suitability of hits against CHIKV.

### 7.4. HSP-90 Inhibitors

Similar to cellular proteins, viral proteins also require chaperone function for folding and assembly. Almost all viruses depend on Hsp90 during their replication [170]. In agreement with this, the Hsp90 inhibitor geldanamycin (GA, 31, Table 2) and its derivatives HS-10 and SNX-2112 were found to reduce CHIKV infection (Table 2) [43,171]. Hsp90 was reported to interact with nsP3 and nsP4 to facilitate CHIKV replication. Recent studies by our group have revealed that treatment with GA reduced new particle formation more effectively in CHIKV S 27, and that Hsp90 is essential at the early stage of CHIKV infection [172]. It was also noted that Hsp90 interacts with and positively regulates nsP2 stability, and inhibitors of Hsp90 can be effective against CHIKV infection [172]. However, development of anti-Hsp90 drugs is not easy. This is because Hsp90 plays critical roles in normal physiology and its inhibition leads to toxicity, including hepatotoxicity and ocular toxicity. Therefore, the clinical utility of Hsp90 inhibitors, including GA and its derivatives, is limited [171]. However, in recent years, second generation Hsp90 inhibitors such as ganetespib have been developed, which are relatively hydrophobic and less toxic [173]. Ganetespib is under clinical trial and its use is yet to be approved. Hence, its efficacy against CHIKV infection is currently not known but will be of interest once the approval is obtained.

### 7.5. Interferon

Interferons (IFN) have attracted considerable attention for the development of antivirals because of their role in innate immune response aftervirus infections [174]. Recombinant IFN-α has been successfully used to treat chronic HCV and HBV infections [175]. Using a similar strategy, CHIKV infection was treated with interferons (IFN-α/β) by Briolant et al. in 2004. Compared to glycyrrhizin, 6-azauridine, and ribavirin, these interferons significantly inhibited CHIKV replication in vitro [82]. Further investigation revealed that the antiviral actions of these interferons in HeLa cells are mediated by induction of the 2′, 5′-oligoadenylate synthetase(OAS) family of proteins [176]. The significance of these interferons against CHIKV infection was also demonstrated in IFN-α/β receptor-deficient mice, in which CHIKV infection caused death [177] due to inadequate IFN-α/β responses inducing hemorrhagic fever and shock [178]. In spite of the interest in interferons as antivirals, relatively little clinical success has been achieved to date [175]. Combination of existing antivirals is an alternate strategy for improving the success of interferon therapy. Recently, a similar strategy was used against CHIKV in vitro by combining interferon with ribavirin [179]. Although the combination showed synergistic antiviral action, further clinical studies are necessary to support its therapeutic utility.

### 7.6. Viperin

Viperin is an interferon-induced host cell protein and is associated with inhibition of viral replication through multiple pathways [180]. Studies show that type I IFNs regulate CHIKV infection via RSAD2 (radical SAM domain-containing 2), which encodes viperin. Mice lacking RSAD2 showed higher CHIKV replication and inflammatory symptoms. Accordingly, viperin has been considered as the host protein capable of reducing CHIKV infection following its localization in the endoplasmic reticulum [181]. Thus, its up-regulation can be used as a strategy to manage CHIKV infection.

### 7.7. Polyinosinicacid:Polycytidylic Acid (poly I:C)

Poly I:C is a potent immune-stimulant and inducer of IFN. Structurally, it resembles double-stranded RNA and stimulates TLR3. Because of this immunomodulation, Poly I:C has long been considered as a potential adjuvant to therapeutic strategies, including those for cancer. However, dissociation of its toxicity from therapeutic efficacy has been a challenge for its clinical application [182]. In recent years, it has been investigated against CHIKV and shown to inhibit CHIKV-induced CPE in human bronchial epithelial cells, as well as virus titers in infected cells. This activity is supposed to be mediated by up-regulation of TLR3, leading to stimulation of IFN and antiviral genes, including *OAS* and *MxA* [171]. Recently, poly I:C-treated mice were shown to have reduced CHIKV titer in brain cells with 100% protection against CHIKV-induced neurological complications. This protection against CHIKV mediated by induction of TLR3, IFN-β, and antiviral genes in mouse brain highlighted its promise as an antiviral agent against CHIKV [183].

### 7.8. Retinoic Acid-Inducible Gene-I (RIG-I) Agonists

Viral nucleic acid activates RIG-I, which, in turn, activates downstream signaling, leading to induction of members of the type I interferon (IFN) family, which are the most important effectors of the innate immune system. Furthermore, RIG-I has been reported to recognize RNA-derived ligands from at least 25 viruses [184]. Accordingly, RNA viruses can be expected to be sensitive to these ligands. Capitalizing on this, Olagnier et al. (2014) showed stimulation of RIG-I in MRC-5 cells by an optimized 5′triphosphorylated RNA molecule, which activated the immune response, leading to reduced virus titer and protection against CHIKV [123]. Since it acts as an immunity booster against CHIKV, RIG-Iis more likely to succeed as an adjuvant to antiviral therapy. However, earlier evasions of RIG-I-mediated antiviral response have been reported in case of RNA viruses, and deregulated RIG-I signaling is associated with autoimmune disorders [185]. Thus, RIG-I activation pathways need to be clearly elucidated to utilize them further against CHIKV.

### 7.9. Repurposing Drugs for Targeting Host Factors of CHIKV

In a recent study, Karlas et al. 2016 [92] attempted to repurpose existing/reported drugs/small molecules against CHIKV by modulating the host factors necessary for CHIKV replication. In the process, they identified 156 proviral and 41 antiviral host factors affecting CHIKV replication. Following screening of drugs against six druggable, proviral targets, including enzymes of fatty acid synthesis (fatty acid synthase, ATP citrate lyase, and acetyl CoA carboxylase), vacuolar-type H^+^ ATPase (vATPase), CDC-like kinase 1 (CLK1), K (lysine) acetyltransferase 5 (KAT5), calmodulin signaling, and fms-related tyrosine kinase 4 (FLT4), they identified 20 small molecule inhibitors with acceptable toxicity. Among these inhibitors, bafilomycin (32, Table 2), which inhibits ATPases, was found to block CHIKV entry. In contrast, drugs, including pimozide (33, Table 2), 5-tetradecyloxy-2-furoic acid (fatty acid synthesis inhibitor) (34, Table 2), cerulenin (35, Table 2), (calmodulin signaling inhibitor), tivozanib (FLT4 inhibitor, 36, Table 2), and anacardic acid (KAT5 inhibitor, 37, Table 2), affected the post-entry stages of CHIKV life cycle. These drugs were found to reduce viral RNA synthesis and inhibit viral release in the supernatants with no detectable cell toxicity. Furthermore, the in vitro results were validated in vivo, in which tivozanib, pimozide, and 5-tetradecyloxy-2-furoic acid showed significant anti-CHIKV properties. The combination of these drugs also exhibited strong anti-CHIKV effects, which indicate its anti-CHIKV potential. 

### 7.10. Protein Duslfide Isomerase (PDI) Inhibitors

Protein disulfide isomerases are folding catalysts, which act as dithiol–disulfide oxidoreductases capable of reducing, oxidizing, and isomerizing disulfide bonds. Since RNA viruses rely upon the host PDI for protein folding and stabilization [186], they offer an alternative way of targeting CHIKV infection via PDI inhibition. Using this strategy, Langsjoen et al. recently evaluated the CHIKV inhibitory effect of PDI-inhibitors, including 16F16 (38, Table 2), PACMA31 (39, Table 2), auranofin (40, Table 2), and EN460 (41, Table 2) [93]. While potent inhibitory activity was observed against CHIKVin vitro, the safety index was not encouraging due to toxicity issues.

## 8. Inhibitors with an Unidentified Target

### 8.1. Lupenone

Lupenone (42, Table 2), a terpenoid phytoconstituent, was first reported with strong viral plaque inhibitory effect against HSV-1 and HSV-2 in 2003 [187]. However, it has not yet attracted attention for further investigation against other viruses. To find novel inhibitors against CHIKV, ethyl acetate extracts of Madagascan plants were screened against CHIKV. Two terpenoids isolated from these extracts, namely, lupenone, and β-amyrone (43, Table 2), showed moderate inhibition of CHIKV replication with EC_50_ of 77 μM and 86 μM, respectively [86]. Owing to moderate antiviral potency and absence of data regarding its mechanism of action and toxicity, further investigations are required before considering it as a hit against CHIKV.

### 8.2. Jatrophan Ester

Jatrophan diterpenes have earlier been reported to possess wide range of medicinal properties [188]. Although some investigations have reported antiviral properties of Jatrophan extracts, relatively little information is available regarding their specific antiviral action [189]. While screening plants from Euphorbia species using a CHIKV cell-based assay, *Euphorbia amygdaloides* subsp. *Semiperfoliata* (Viv.) A. R. Sm. was found to possess the most potent anti-CHIKV activity. Accordingly, bioassay-guided purification of an ethyl acetate extract of entire *Euphorbia amygdaloides* subsp. *semiperfoliata* was performed, which led to the identification of new jatrophan esters (44, Table 2) as potent inhibitors of CHIKV [94]. Because of structural similarities with other diterpenoid esters that are known activators of PKC, a PKC-mediated mechanism was proposed for the antiviral effect of these compounds against CHIKV, which, however, awaits experimental validation. Considering the high molecular weight (592.76) and violation of Lipinski’s rule of five, in vivo availability should be assessed while evaluating efficacy in animal models. Since analogues of these esters also show antiviral effects against CHIKV, there is scope for further structural modification of the hit molecules for optimizing in vivo efficacy.

### 8.3. Trigocherrierin A and B

Daphnane-type diterpenoids of Trigonostemon have been reported to possess anti-HIV-I properties [190]. Thus, to identify new hits against CHIKV, an ethanol extract of the leaves of *Trigonostemoncherrieri* was screened, leading to the identification of highly oxygenated daphnane diterpenoid orthoesters (DDO) that had an uncommon chlorinated moiety. These trigocherrins exhibited anti-CHIKV property, with EC_50_ ranging from 0.6 to 3.9 μM. Among these compounds, trigocherrierin A (45, Table 2) and B (46, Table 2) were identified as potent diterpenoids with EC_50_ of 1.5 μM and 2.6 μM, respectively, against CHIKV in a virus cell-based assay [95,126]. With a good safety index, these compounds could be hits against CHIKV. However, further studies are required to determine their mode of action and efficacy before optimization as leads against CHIKV.

### 8.4. Extract of Hyptis Suaveolens

In a random screening of plant species against CHIKV, the aqueous ethanolic extract of leaves of *Hyptis suaveolens* has been shown to be selectively potent against an Asian strain of CHIKV with an EC_50_ of 15.62 μM [191]. The basis of this selectivity is not clear. Pentacyclic triterpenoids are the major constituents of this extract, which possibly contribute to the antiviral properties of diterpenoids. However, the antiviral action against CHIKV at present cannot be directly attributed to these terpenoids without further investigation. 

8.5. 5,7-Dihydroxyflavones

During the screening of natural compounds and clinical drugs, Pohjala et al. identified that 5,7-dihydroxyflavones, including apigenin (47, Table 2), chrysin, naringenin, and silybin, suppress activities of *GFP* and luc marker genes expressed by the CHIKV replicon [69]. Apigenin (CC_50_: >200 μM) possesses good antiviral action against CHIKV with EC_50_ value in the range of 22.5 μM to 28.3 μM [69]. Recently, apigenin, along with luteolin from the ethanolic extract of *C. dactylon*, showed antiviral properties against CHIKV at minimal concentrations (25 to 50 μg/mL) without any cytotoxicity [192]. However, the mechanism of this antiviral action remains to be elucidated. Nonetheless, apigenin can be considered as a good hit against CHIKV due to its additional benefits against inflammation. In addition, owing to its ability to modulate drug metabolism and reverse drug resistance, it can be considered as a co-drug with other antivirals. 

### 8.6. Triazolopyrimidines

Through a collaborative research effort, Gigante et al. identified 1-[3-(7-hydroxy-5-methyl-3*H*-[1,2,3] triazolo [4,5-d] pyrimidin-3-yl) phenyl] ethanone as a hit against CHIKV while screening a structurally diverse group of compounds [151]. Only few reports regarding antiviral properties of triazolopyrimidines exist, and the hits were randomly modified to develop structural analogues. Further, the evaluation of these analogues against CHIKV replication in Vero cells revealed 5-ethyl-3-(3′-isopropoxyphenyl)-3H-[1,2,3] triazolo [4,5-d]-pyrimidin-7(6H)-one [48, Table 2] as the most potent compound with high (>200) safety index [124]. The inhibitory activity was also found to be specific for CHIKV. Accordingly, the target of this compound is considered to be CHIKV-specific, which, however, is yet to be identified. This compound does not violate Lipinski’s rule of five and hence can be considered for further in vivo evaluation to establish its effectiveness.

### 8.7. Benzouracil-Coumarin-Arene-Conjugates

Uracil derivatives have been reported earlier to inhibit viruses, including HIV-I and HBV [193,194]. Antiviral actions have also been reported for coumarin compounds [195]. Although mere hybridization of this class of compounds cannot ensure antiviral properties, Hwu et al. designed and developed 22 structural conjugates of uracil, coumarin, and arene for evaluation against CHIKV on the basis of structural analysis of these compounds. Five of the conjugates exhibited significant potency (EC_50_: 10.2–19.1 μM) against CHIKV [96]. Coumarin was suggested to be critical for the antiviral action against CHIKV using structure-activity analysis. However, the SI (CC_50_/EC_50_) of these compounds was less than 12, which limits their further consideration. Additionally, the conjugate,2-oxo-4-([(4-oxo-3,4-dihydroquinazolin-2-yl)thio]methyl)-2H-chromen-7-yl-4-methylbenzenesulfonate (49, Table 2) showed SI of 11.5 and can be considered for further evaluation to determine the mode of action.

### 8.8. Thiazolidinone Derivatives

Thiazolidinones constitute one of the important classes of compounds with inhibitory properties against microbes, fungi, and viruses [196]. In a recent study, a series of arylalkylidene derivatives of thiazolidinones were synthesized and screened against CHIKV (LR2006_OPY1) in Vero cell culture using the CPE reduction assay (Table 2) [97]. Five of these derivatives showed the potential for CHIKV inhibition. The compound, 5-(2-methylphenyl)-methylidene)-2-sulfanylidene-1, 3-thiazolidin-4-one (50, Table 2), exhibited the highest inhibitory properties with good SI (IC_50_: 0.42 μM, CC_50_: > 100 μM). In silico studies have suggested its ability to inhibit nsP2; however, it is difficult to confirm its mechanism of CHIKV inhibition without further experimental details. Nevertheless, based on in vitro potency against CHIKV and good drug likeness, it can be considered as an important lead candidate for further optimization in drug development against CHIKV.

### 8.9. MBZM-N-IBT

Isatin-β-thiosemicarbazone and benzimidazole derivatives were earlier known to be effective against some viruses; however, reports regarding their efficacy against CHIKV or other alphaviruses were limited. Our group screened MBZM-N-IBT (51, Table 2) as a conjugate of these molecules against CHIKV. The EC_50_ value against CHIKV (S27 and DRDE-06) was 38.68 and 58.33 μM, whereas CC_50_ was greater than 800 μΜ. Viral RNA synthesis was reduced by 65.53% and 23.73% for nsP2 and E1, respectively. Interestingly, it reduced viral protein levels by 95% for both nsP2 and E2 inhibition. Further, it was effective with a very short exposure time of 1hpi, both at early and late stages of the CHIKV life cycle, suggesting its involvement in multiple pathways of CHIKV inhibition. Although in silicostudies suggest its ability to directly inhibit both structural and non-structural proteins, further investigations are required to understand its exact mode of action. However, with good drug-like property predictions for MBZM-N-IBT, it is a potential lead candidate against CHIKV [52].

### 8.10. Berberine, Abamectin, rough a collaborative research effort, Gigante et al. identifieand Ivermectin

In a high throughput screening of approximately 3000 compounds, Varghese et al. identified abamectin (52, Table 2), ivermectin (53, Table 2), and berberine (54, Table 2) with EC_50_ of 1.5, 0.6, and 1.8 μM, respectively, against CHIKV [98]. Abamectin and ivermectin are macrocyclic lactones with broad-spectrum antiparasitic action. Along with berberine, a plant-derived isoquinoline alkaloid, it inhibited CHIKV in a dose-dependent manner. These compounds also exhibited antiviral action against other alphaviruses, including SFV and Sindbis virus. Although the exact modes of action of these compounds are not well understood, it was speculated that viral replication might be reduced by abrogating the synthesis of CHIKV genomic and sub-genomic viral RNA, as well as down-regulating viral protein level. Considering the wide range of bioactivities exhibited by berberine [197], its interference with certain host factors required for CHIKV replication cannot be ruled out. In addition, it was found to be more effective than abamectin and ivermectin in reducing the number of infectious particles produced, which indicated that berberine interferes in the later phase of the CHIKV infection cycle, which, however, requires further investigation [98].

### 8.11. 5-Chloro-N-{4-[(1E)-1-{2-[(2-phenylcyclopropyl) carbonyl] hydrazinylidene} ethyl] phenyl} Thiophene-2-carboxamide

Baseto et al. have earlier identified (2E)-3-(4-tert-butylphenyl)methylidene] prop-2-enehydrazide as a potential hit and predicted its capacity to inhibit nsP2 protease based on molecular docking studies [60]. While it considerably reduces CHIKV infection, a recent study demonstrated it to be a poor inhibitor of nsP2 protease activity [62]. Nevertheless, Das et al. has used this for further optimization and screened twelve molecules against CHIKV [62]. About 75% of these molecules inhibited the protease activity of nsP2 at a concentration of 200 µM. Molecules that inhibited protease activity of nsP2 also inhibited CHIKV replication with EC_50_ < 50 µM. All compounds exhibited CC_50_ > 200 µM. Compound 55 (Table 2) was the most potent inhibitor of CHIKV replication (Table 2) with an EC_50_ of 1.5 µM and SI of more than 133 [62]. However, it showed relatively low nsP2 protease inhibition. Furthermore, at concentrations lower than 50 µM, it could not significantly inhibit CHIKV positive strand RNA synthesis. Accordingly, its CHIKV inhibitory capacity can be ascribed to cell-mediated mechanisms or other modes of action which are yet to be elucidated. However, considering potency and SI, it can be further screened in pre-clinical models to establish its potential as a possible antiviral candidate against CHIKV.

### 8.12. Compound ID1452-2 

Luciferase gene expression was shown to be inhibited in the presence of CHIKV nsP2 [99]. Accordingly, this transcriptional assay was used as a screening tool, which led to the identification of ID1452-2 (56, Table 2) as a hit compound that alleviated nsP2-mediated shutoff on-gene transcription, leading to restoration of luciferase expression [198]. Although, the mode of interference with nsP2 is yet to be elucidated, this assay can be useful for large-scale screening of hits against nsP2.

## 9. Pre-Clinical Validation of Molecules

Approved drugs that have been studied in vitro for repositioning as antivirals for CHIKV can be subjected to direct clinical studies against CHIKV infection. However, clinical studies so far have failed to identify an effective antiviral for therapeutic management of CHIKV. In this scenario, molecules that have shown enough in vitro potential against CHIKV should be further validated in pre-clinical models. SI should be considered while selecting molecules for pre-clinical trials. Owing to the existence of polypharmacology in vivo, the SI calculated by in vitro studies may not guarantee safety. In addition, there is no definite cutoff value for SI in in vivo studies. Nonetheless, a value ≥10 can be considered as a cutoff for studies in animal models. 

The C57BL/6 model has been widely used for studies on CHIKV infection. However, these mice lack signs of polyarthralgia and chronic inflammation, which are common symptoms in humans, following CHIKV infection [199]. Although, adult cynomolgus macaques have been shown to manifest chronic arthralgia like humans, their use has been rare because of cost and ethical issues [200]. In this scenario, humanized mice can be explored as a model, as they have been used previously for studying a wide range of human pathogens, including HIV-1, Epstein Barr virus (EBV), and HCV [201]. However, this is still at an early stage and requires further optimization for in vivo recapitulation of CHIKV disease symptoms. In this scenario, the C57BL/6 model can still be considered as the primary model of CHIKV infection. Thus, the molecules must be screened in this model for their effectiveness in terms of reducing viremia, histological damage, foot-pad inflammation, and other immunohistochemistry parameters.

## 10. CHIKV Coinfection

CHIKV and DENV are transmitted by the same *Aedes* spp. mosquitoes. Thus, there are increasing rates of reports of their coinfection [15,16,17]. Since the clinical symptoms are similar in nature, their diagnosis based on symptoms alone is difficult. ZIKV infection also shows similar clinical symptoms, and it is transmitted by *Ae. aegypti* mosquitoes, which also act as vectors of CHIKV and DENV. As both CHIKV and ZIKV can be transmitted simultaneously in a single bite of this mosquito, it is reasonable to expect their coinfection [202]. Besides, multiple coinfections with DENV, CHIKV, and ZIKV are also possible [16]. Furthermore, coinfection has been shown to facilitate faster replication in *Ae. Aegypti,* and a similar phenomenon in humans can aggravate the infection [203]. In this scenario, finding antivirals to manage the coinfections is a challenge. Incidentally, there is no specific antiviral against these entire viruses. Hence, hits identified against CHIKV also need to be tested against these viruses. While some of the hits against CHIKV including chloroquine [204], 6-azauridine [205], ribavirin [205], silymarin [206], and suramin [207] have also shown inhibitory potential against DENV, recently, EGCG was shown to inhibit entry of Zika virus [208].Thus these hits should be preferred for investigation in coinfection models in vitro. However, the in vivo investigation of these hits for further development is a challenge, as the disease pathology of coinfection is not clearly known. Hence, the immediate challenge is to validate coinfection models to verify the in vivo antiviral efficacy of these molecules.

## 11. Conclusions

Finding effective antivirals for CHIKV has not been easy. The repurposing of existing drugs, including chloroquine, arbidol, chlorpromazine, imipramine, and mefenamic acid has achieved only limited success. Most of them inhibit CHIKV entry with questionable therapeutic effectiveness. Accordingly, efforts have been made to directly target CHIKV replication byinhibiting the CHIKV genome. Several molecules, including ribavirn, flavipiravir, 6-azaurdine, suramin, silymarin, mycophenolic acid, and andrographolides, have been investigated. Among these molecules, only ribavirin has been studied in some detail and appears to be the most eligible candidate for further investigation on account of its clinical acceptance as an antiviral and ability to synergize antiviral effects with molecules that have different modes of action, such as NSAIDs and interferons.

Targeting host factors involved in viral replication is an alternative strategy. Accordingly, molecules known to inhibit furin, protein kinase, and Hsp90 have been shown to inhibit CHIKV replication in vitro. Further, modulators of PKC and interferons can inhibit CHIKV replication in vitro. However, validation of these observations in pre-clinical studies isessential to support these findings. In addition, the host targets and their isoforms are critical for normal physiological function. Hence, the specificity and selectivity of these molecules for host targets and their isoforms have to be determined. The in vivo model for the further evaluation of hits should consider the above factors prior to developing an effective antiviral.

Relatively few molecules have been shown to directly inhibit CHIKV proteins. Although harringtonine and homoharringtonine target CHIKV protein translation, whether they directly affect CHIKV proteins is still unknown. This is mostly due to the slow progress in the purification and elucidation of these target proteins. This also has limited rational drug design and high throughput screening of libraries for identification of hits. However, the purification of nsP2 protease and the elucidation of its structure have fuelled some recent research towards determination of potent hits against CHIKV. Many hits have been identified using in silico approaches considering the structure of nsP2 protease as a target. However, these hits have to be experimentally validated against specific targets, which can further lead to pharmacophore mapping for the development of more potent inhibitors. Additionally, other approaches can be used to find specific hits against CHIKV proteins. One such approach is developing Zn-finger inhibitors, which has earlier been demonstrated against other viruses [209,210]. So far, Zn binding domains have been identified in several alphaviruses [47]. The PDB structure of CHIKV (S 27) has His-X-X-X-His and Cys-X-X-X-Cys consensus motifs, which can co-ordinate Zn in several important proteins of CHIKV, including nonstructural polyproteins, and immature and mature envelope glycoprotein complexes. Therefore, the targeting of these sites can be a possible option for inhibiting CHIKV.

The hit to lead progress against CHIKV has been poor. This is due to difficulties in finding a suitable model of CHIKV infection. The C57BL/6 mouse model has been generally used to reproduce symptoms exhibited by infected humans, including self-limiting arthritis, myositis, and tenosynovitis. However, the immune response can vary with the age of mice and CHIKV strain used for inducing infection. In addition, the response to CHIKV infection can be different for humans and mouse models. Therefore, an animal model with an immune system that is more genetically similar to that of humans should be used for better reproducibility of infection conditions [199]. Although these humanized mouse models are still exploratory, they can be used along with C57BL/6 models to complement the findings regarding the effects on CHIKV infection and immunity.

Thus, while an effort to develop new hits against rational experiments should be continued further, investigations of in vivo pathogenesis are warranted to further support strategies for the regulation of CHIKV infection.

## Figures and Tables

**Table 1:** Compounds identified as CHIKV inhibitor by in silico screening.

Sl No.	Compound	Structure	Target	References
1	N-butyl-9-[3,4-dipropoxy-5-(propoxymethyl) oxolan-2-yl]purin-6-amine	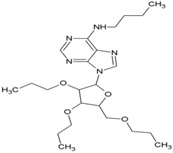	nsP2 Protease	[56]
2	ASN 01541696	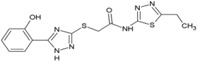	nsP2 Protease	[59]
3	(2E)-3-(4-tert-butylphenyl) methylidene]prop-2-Enehydrazide	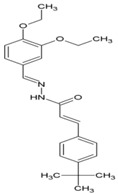	nsP2 Protease	[60]
4	NCL 61610	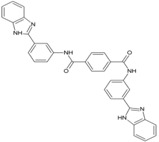	nsP2 Protease	[63]
5	CID_5808891	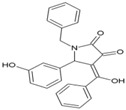	nsP2 Protease	[64]
6	ZINC67680487	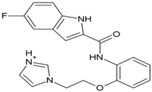	nsP2 Protease	[65]
7	ZINC04725220	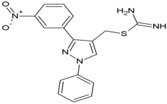	nsP2 Protease	[54]
8	Doxycycline	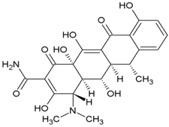	nsP2, E2	[66]
9	BILN2106	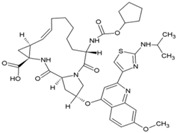	nsP4	[67]
10	JTK 109	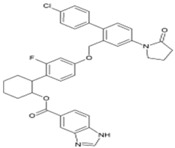	nsP4	[67]
11	Baicalin	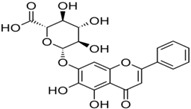	nsP3	[68]
12	Quercetagetin	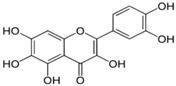	nsP3	[68]
13	Naringenin	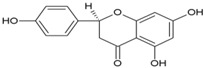	nsP3	[69]
14	Picolinic acid	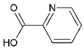	Capsid protein	[70]

**Table 2:** Compounds with anti-CHIKV action.

Sl No.	Compound	Structure	EC_50_	CC_50_	Reference
Compounds interfering with CHIKV internalization					
1	Chloroquine	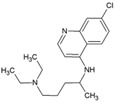	17.2 µM	260 µM	[71]
2	Arbidol/Umifenovir	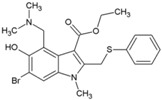	12.2 µM	376 µM	[72,73]
*3*	*tert*-Butyl 5-(hydroxymethyl)-1-methyl-2-((2,6-dichloro phenyl sulfynyl)methyl)-1H-indol-3-carboxylate	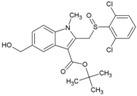	30 ± 4 µM	397 ± 24 µM	[74]
*4*	*tert*-Butyl 5-(hydroxyl methyl)-1-methyl-2-((2-trifluoromethyl phenyl sulfynyl) methyl)-1H-indol-3-carboxylate	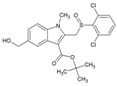	32 ± 1.1 µM (Vero cells)	>468 µM (MTS/PMS)	[74]
5	Chlorpromazine	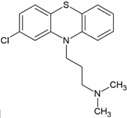	NR	NR	[69]
6	EGCG	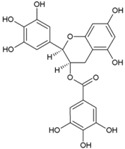	NR	NR	[75]
7	FL3(Flavagline)	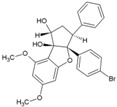	22.4 nM(HEK293T)	118.77nM(MTT)	[76]
8	Mefenamic acid	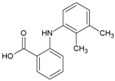	13μM(VeroE6)	>100 μM(MTT)	[66]
9	Meclofenamic acid	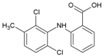	18 μM(VeroE6)	>100 μM(MTT)	[66]
10	U18666A	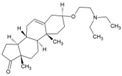	NR	NR	[77]
11	Imipramine	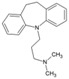	NR	NR	[77]
12	Curcumin	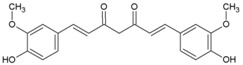	3.89 μM(Hela, BHK21, VeroE6)	11.6 μM(Trypan blue)	[78]
13	Demethoxycurcumin	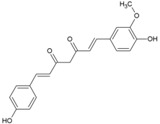	0.89μM (Hela, BHK21, VeroE6)	13.2 μM(Trypan blue)	[78]
Compounds inhibiting CHIKV genome replication					
14	Andrographolide	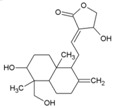	77 µM	1098 µM (MTT Almarblue assay),	[79]
15	Ribavirin	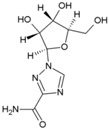	341.1 µM	30.7 mM	[80]
16	Mycophenolic acid	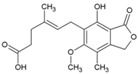	0.1 µM (VeroE6)	30 µM (MTT)	[81]
17	6-Azauridine	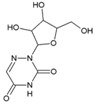	0.2 μg/mL (VeroE6)	51 μg/mL (Trypan blue)	[82]
18	Favipiravir	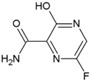	5.9 ± 3.3 µM (VeroE6)	NR	[83]
19	T-1105	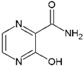	2.8 ± 0.3 (Vero E6)	NR	[83]
20	Suramin	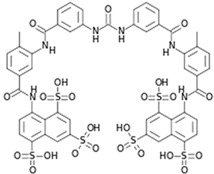	8.8–28.9 µM (VeroE6, BHK21)	700 µM (MTS)	[84]
Compounds inhibiting CHIKV protein translation					
21	Harringtonine	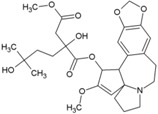	0.24 µM (BHK21)	NR	[85]
Compounds targeting host factors to inhibit CHIKV					
22	dec-RVKR-cmk	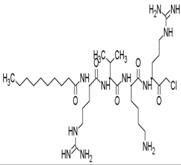	NR	NR	[62]
23	Prostratin	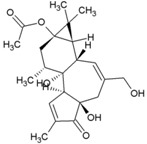	5.7µM (VeroE6)	NR	[86]
24	12-*O*-tetradecanoylphorbol 13-acetate	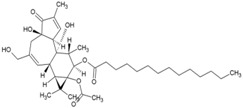	2.9 nM (Vero Cells)	5.7 µM (MTT)	[86]
25	12-*O*-decanoyl-7-hydroperoxy-phorbol-5-ene-13-acetate	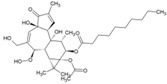	NR	NR	[87]
26	Debromoaplysiatoxin	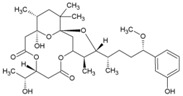	1.3 µM (STCRH30)	13.9 µM (Almar Blue)	[88]
27	3-methoxy debromoaplysiatoxin	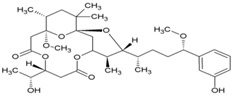	2.7 µM (STCRH30)	24.8 µM (AlmarBlue)	[88]
28	Phorbol-12, 13-didecanoate	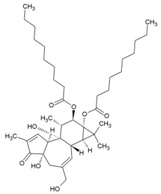	6.0 ± 0.9 μM	NR	[89]
29	Bryostatin-21	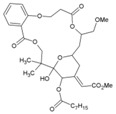	2.2 μM	>50 μM	[90]
30	2-(1-hydroxy-2-methylpropyl)-*N*-[4-(propan-2-yl)phenyl]-1,3-thiazole-4-carboxamide	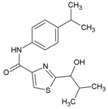	2.2 µM (HuH7)	>50 µM (Resazurin)	[91]
31	Geldanamycin	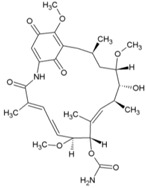	NR	NR	[43]
32	Bafilomycin	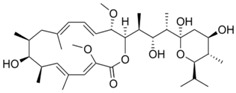	0.33 nM (HEK293T	0.003 µM (WST-1 assay)	[92]
33	Pimozide	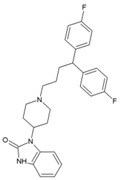	0.28 μM(HEK293T)	19.18 μM (WST-1 assay)	[92]
34	5-tetradecyloxy-2-furoic acid	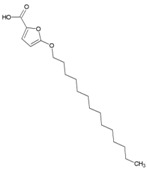	0.15 μM (HEK293T)	>60 μM(WST-1 assay)	[92]
35	Cerulenin	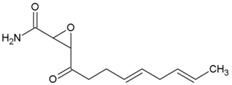	3 µM (HEK293T)	7.57 µM (WST-1 assay)	[92]
36	Tivozanib	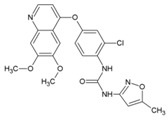	0.8 μM (HEK293T)	8.34 μM(WST-1 assay)	[92]
37	Anacardic acid	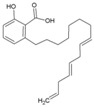	0.58 μM (HEK293T)	2.68 μM(WST-1 assay)	[92]
38	16F16	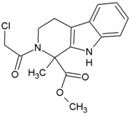	6.6 μM (HEK293T)	8.9 μM (Almarblue assay)	[93]
39	PACMA31	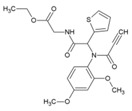	12.1 μM (HEK293T)	12.2 μM (Almarblue assay)	[93]
40	Auranofin	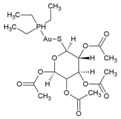	27.0 μM (HEK293T)	31.1 μM (Almarblue assay)	[93]
41	EN460	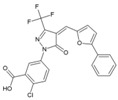	1.0 μM (HEK293T)	1.6 μM (Almarblue assay)	[93]
Compound with unknown CHIKV target					
42	Lupenone	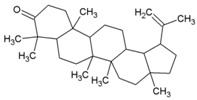	77 µM (Vero)	>235 µM	[86]
43	β-amyrone	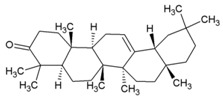	86 (Vero)		[86]
44	Jatropha ester	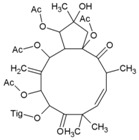	0.76 ± 0.14 µM	159 µM	[94]
45	Trigocherrin A	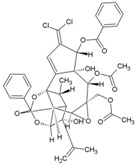	1.5 ± 0.6 µM (VeroE6)	35 ± 8 µM	[95]
46	Trigocherrin B	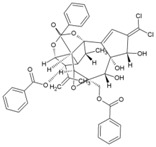	2.6 ± 0.7 µM (VeroE6)	93 ± 3 µM	[95]
47	Apigenin	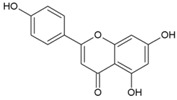	70.8 µM	>200 µM	[69]
48	5-Ethyl-3-(3′-isopropoxyphenyl)-3H-[1,2,3] triazolo [4,5-d]-pyrimidin-7(6H)-one	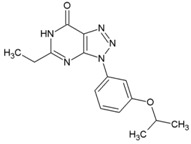	3 ± 1 µM (VeroE6)	>668 µM	[38]
49	2-Oxo-4-([(4-oxo-3,4-dihydroquinazolin-2-yl)thio]methyl)-2H-chromen-7-yl4-methylbenzenesulfonate	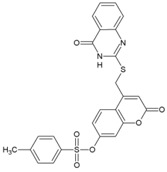	10.2 µM (VeroE6)	117 µM	[96]
50	5-[(2-Methylphenyl)-methylidene]-2-sulfanylidene-1,3-thiazolidin-4-one	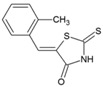	0.42 μM(VeroE6)	>100 μM	[97]
51	MBZM-N-IBT	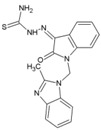	38.68 µM (S27),58.33 µM (DRDE-06) (VeroE6)	>800 μM	[52]
52	Abamectin	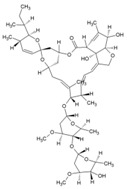	1.5 ± 0.6 µM (BHK21)	28.2 ± 1.1 µM	[98]
53	Ivermectin	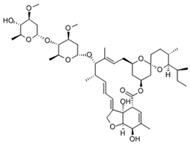	0.6 ± 0.1 µM (BHK21)	37.9 ± 7.6 µM	[98]
54	Berberin	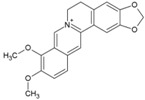	1.8 ± 0.5 µM (BHK21)	>100 µM	[98]
55	5-chloro-*N*-{4-[(1E)-1-{2-[(2-phenylcyclopropyl) carbonyl] hydrazinylidene} ethyl] phenyl} thiophene-2-carboxamide	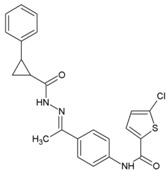	1.5 μM	>200 μM	[62]
56	ID1452-2	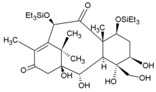	NR	NR	[99]

NR: Not reported, MTT: 3-(4,5-dimethylthiazol-2-yl)-2,5-diphenyltetrazolium bromide, MTS: 3-(4,5-dimethylthiazol-2-yl)-5-(3-carboxymethoxyphenyl)-2-(4-sulfophenyl)-2*H*-tetrazolium, WST: water-soluble tetrazolium salts.
